# Acupuncture for chronic, stable angina pectoris and an investigation of the characteristics of acupoint specificity: study protocol for a multicenter randomized controlled trial

**DOI:** 10.1186/1745-6215-15-50

**Published:** 2014-02-05

**Authors:** Dehua Li, Mingxiao Yang, Ling Zhao, Hui Zheng, Ying Li, Xiaorong Chang, Jin Cui, Ruihui Wang, Jing Shi, Junling Lv, Junyan Leng, Juan Li, Fanrong Liang

**Affiliations:** 1 Chengdu University of Traditional Chinese Medicine, Chengdu, Sichuan, China; 2 Hunan University of Traditional Chinese Medicine, Changsha, Hunan, China; 3 Guiyang College of Traditional Chinese Medicine, Guiyang, Guizhou, PR China; 4 Yunnan Provincial Hospital of Traditional Chinese Medicine, Kunming, Yunnan, China; 5 Shanxi College of Traditional Chinese Medicine, Xi’an, Shanxi, China; 6 School of Acupuncture and Tuina, Chengdu University of Traditional Chinese Medicine, No.37, Shi Er Qiao Road, Jinniu District, Chengdu 610075, China

**Keywords:** Chronic stable angina pectoris, Acupuncture, Acupoint specificity, Randomized controlled trial

## Abstract

**Background:**

Chronic stable angina pectoris (CSAP) is a common cardiovascular condition that endangers a patient’s life quality and longevity. As demonstrated in several clinical trials, acupuncture is attested to be effective for CSAP. Current trials are not adequate enough to provide high-quality evidence for clinical decision making, as a result of inadequate methodology design and small sample size. Notably, stark controversy toward acupoint specificity also exists in the clinical acupuncture trials for CSAP. Therefore, we designed the present study as a randomized controlled trial primarily to investigate the effectiveness of acupuncture in addition to routine care among patients with CSAP. Meanwhile, we examined whether acupoint on the disease-affected meridian (DAM) is superior to either acupoint on the non-affected meridian (NAM) or non-acupoint (NA), to further investigate the meridian-based characteristics of acupoint specificity.

**Methods/Design:**

This study was a multicenter, assessor and statistician blinded, randomized controlled trial in China. In this study, 404 participants in sum will be randomly assigned to four groups through central randomization in a 1:1:1:1 ratio. The whole study period is 20 weeks including a 4-week baseline period, a 4-week treatment period and a 12-week follow-up. Participants in the DAM group receive acupuncture stimulation at acupoints on the disease-affected meridian, and three different control groups will undergo acupuncture stimulation at the NAM, the non-acupoint and no intervention respectively, in addition to basic treatment. Participants in the acupuncture groups will receive 12 sessions of acupuncture treatment over 4 weeks, while the wait-listed (WL) group would receive free acupuncture treatment after the completion of the study. The outcome measures in this trial include the frequency of angina attack during 4 weeks as the primary outcome and eight other secondary outcomes.

**Discussion:**

This trial will provide new and relatively high-quality evidence in acupuncture treatment for CSAP. Moreover, this trial may further validate the meridian-based characteristics of acupoint specificity by comparing the strength of acupoints on the disease-affected meridian versus that of the non-affected meridian, to further inspire optimization of acupuncture therapy for CSAP.

**Trial registration:**

Clinical Trials.gov NCT01686230

## Background

Chronic stable angina pectoris (CSAP) is a common cardiovascular condition that endangers a patient’s life quality and longevity, which is characterized by severe chest pain and discomfort in the left anterior chest or adjacent areas caused by myocardial ischemia [1]. Stable angina affects more than 7.8 million people in the United States, with an annual incidence of over 500,000 new cases [2]. Despite the declining incidence of myocardial infarction, the prevalence of angina remains high, and direct costs in the United States in 2000 have been estimated at up to $75 billion [3]. As for China, the prevalence of angina is 2.4% among males and 3.2% among females [4], which makes it a serious social problem, considering its large population base. In China, a large majority of CSAP patients resort to acupuncture and other traditional Chinese medicine (TCM) therapies in addition to conventional drugs for treatment and recurrence prevention [5,6], though discrepancies still exist concerning the effectiveness and efficacy of acupuncture therapy for angina.

Acupuncture, well known as an oriental healing technique that originated from ancient China, has been used as a treatment method in Asia for over 2,000 years. Nowadays, the therapeutic effect of acupuncture is gradually being recognized in the western world. The National Institutes of Health (NIH) Consensus has recommended acupuncture as an alternative and complementary treatment for many health conditions [7]. As demonstrated in several international clinical trials 20 years ago, acupuncture is effective for CSAP [8] in reducing disease duration [9], anginal attack and nitroglycerin consumption [10], as well as for improving cardiac work capacity [11]. Similarly, clinical trials [12,13] and case observations [14-17] from China in recent decades, accompanied with TCM experts’ opinions [18], have consistently confirmed that CSAP patients may benefits from traditional acupuncture therapy. Importantly, large amount of animal experiments have already validated the myocardial protective effect of acupuncture for cardiac ischemia [19,20] and reperfusion via inhibition of the beta(1)-adrenoceptor signaling pathway [21,22] and regulation of myocardial enzyme level [23,24]. However, these Chinese clinical trials or observations and international randomized controlled trials (RCTs) are not adequate enough to act as high-quality evidence for clinical decision making, as a result of inadequate methodology design and small sample size [25]. Therefore, clinical trials with sufficient sample size and sound methodology design are necessary and meaningful to clinical practice.

Notably, there is a remarkable paradox in the aforementioned international clinical trials validating the effectiveness of acupuncture for CSAP, which is the specificity of real acupoint when compared with sham acupoint [8-11]. In TCM theory, multifaceted factors contribute to therapeutic effect of acupuncture [26], among which, the selection of optimal acupoint is vital. As to acupoint selection, the traditional acupuncture theory emphasizes the indications and property of different acupoints, which is known as acupoint specificity [27]. Acupoint specificity has been widely acknowledged and considerably utilized as the basic law of traditional acupuncture practice. To elaborate, acupuncturists would always prefer to choose acupoints on the disease-affected meridian(s) for preferable treatment effect based on TCM patterns resulting from syndrome differentiation [28]. Nevertheless, many reviews, meta-analyses and clinical trials have merely demonstrated a statistical difference, but not clinical significance between real acupoint and sham point for various diseases [29-36]. These studies have drawn the attention of many researchers and further aroused controversy regarding the existence of acupoint specificity. In 2010, the American Association of Acupuncture came to a consensus agreement and announced in a white paper that acupoint specificity was one of these two main paradoxes of forthcoming acupuncture research [28]. Hence, clinical trials assessing the meridian-involved acupuncture specificity is of great significance for guiding clinical practice as well as for inspiring basic research in acupuncture.

Therefore, we designed the present study as a randomized controlled trial to primarily investigate the effectiveness of acupuncture, in addition to routine care, among patients with CSAP. Meanwhile, we examined whether acupoint on the disease-affected meridian (DAM) is superior to either acupoint on the non-affected meridian (NAM) or non-acupoint (NA), to further investigate the meridian-based characteristics of acupoint specificity. The present study was financially supported by the National Basic Research Program (973 Program) in China and the trial was registered with an identifier (NCT01686230) in Clinical Trials.gov.

## Methods/Design

### Ethics review and informed consent

The study protocol was reviewed and approved by the IRB of the Teaching Hospital of Chengdu University of Traditional Chinese Medicine (No.2012KL-005). The design of this trial is in accordance with the Declaration of Helsinki (Version Edinburgh 2000). Before randomization, all eligible patients will be informed of the details of the study and all the benefits and risk that they may take from this trial. Particularly, participants will be clearly told about the equal chance of allocation to any one of the four groups before signing the informed consent. Meanwhile, they will be given enough time to decide whether they join in the trial or not. Lastly, participants will be included voluntarily by signing the written informed consent. However, due to the principle of blinding, only patients allocated to WL group will be told to wait for free treatments till the completion of study; while allocation information will be strictly restrained to patients in the other three groups.

### Design

**Figure 1:**
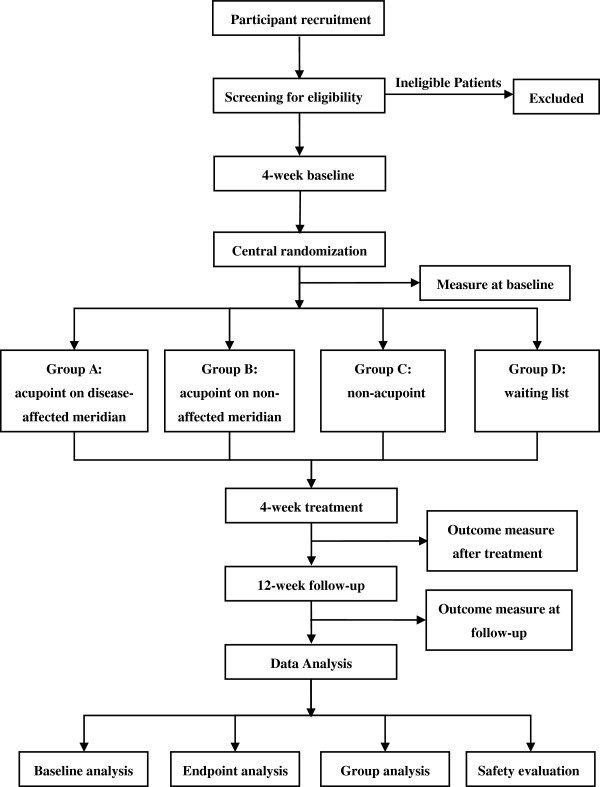
Trial flow chart.

This study was a multicenter, assessor and statistician blinded, randomized controlled trial in China. In this study, 404 participants in sum would be randomly assigned to the four groups through central randomization in a 1:1:1:1 ratio (Figure 1, Table 1). Eligible participants will be recruited from outpatient clinics and inpatient departments of Cardiology in the following five clinical centers in different regions of China: Chengdu University of TCM, Hunan University of TCM, Yunnan College of TCM, Guiyang College of TCM and Shanxi College of TCM.

**Table 1:** Trial process chart

**Period**	**Baseline**	**Inclusion**	**Treatment**	**Follow-up**		
**Measurement**		1	2	3	4	5
**Week**	-4	0	4	8	12	16
**Patients**						
Informed consent						
Inclusion/exclusion criteria	×	×				
Medical history	×					
Medical examination	×					
Combined disease treatment	×	×	×	×	×	×
**Physical examination**	×					
**Outcomes**						
Angina diary	×	×	×	×	×	×
The frequency of angina attack	×	×	×	×	×	×
The dosage of nitroglycerin	×	×	×	×	×	×
Angina pectoris grade		×	×	×	×	×
The pain severity of angina(VAS)	×	×	×	×	×	×
SAQ score		×	×	×	×	×
Six minutes’ walk test		×	×			
SAS and SDS		×	×	×		×
24 hours dynamic ECG		×	×			
Cardiovascular events			×	×	×	×
**Trial evaluation**						
Patient’s compliance			×			×
Reasons of drop-out or withdrawals			×			×
Adverse events						×
Safety evaluation						×

ECG, dynamic electrocardiograph; SAQ, Seattle Angina Questionnaire; SAS, Self-Rating Anxiety Scale; SDS, Self-Rating Depression Scale; VAS, Visual Analogue Scale.

### Randomization

The central randomization is performed by the Company of Brightech-Magnsoft Clinical Information Management System (CIMS). Allocation to the treatment groups uses a stratified block dynamic randomization method with permuted block, which is automatically under the control of a central computer system. The website and mobile messages will be used to send randomization information (including the participants’ name in pinyin format, gender and date of birth) to the center. To guarantee allocation concealment, randomization will be done by an independent researcher. An independent assessor will interview the participants and perform the screening. Random numbers and group assignment will be confirmed through email or short message service (SMS) to the independent assessor immediately. This procedure guarantees that randomization concealment is adequate, and not influenced either by the acupuncturists or by the participants. Participants allocated to groups will be blinded to their treatment allocation; however blinding is clearly not possible in the WL control group. We shall endeavor to ensure that participants begin the trial with the same expectations of effectiveness by informing them that all the treatments provided are effective. All participants will be assessed and the results analyzed by professionals blinded with respect to the allocations of the different treatments.

### Participants

#### Study population

This trial focused on the patients with CSAP. Eligible patients should match the inclusion criteria; ineligible patient should be excluded according to the exclusion criteria.

### Inclusion criteria

Participants will be included if they fulfill the following criteria:1) patients meet the diagnostic criteria of ACC/AHA angina pectoris of coronary heart disease [37]; 2) 35 ≤ age ≤80 years, male or female; 3) the onset of angina pectoris ≥3 months previous and the frequency of angina attack ≥ twice a week; and 4) patients signed the informed consent.

### Exclusion criteria

Patients with any of the following conditions will be excluded: 1) age ≤35 or age ≥80; 2) pregnant or lactating women; 3) have complicated cardiovascular, digestive, respiratory, urinary, blood, nervous, endocrine system and other severe primary diseases and failed to effectively control in clinic; 4) have psychiatric, allergic or blood disorders; 5) have acute coronary syndrome (including acute myocardial infarction and unstable angina), severe arrhythmias (severe atrioventricular block, ventricular tachycardia, heartbeat influencing the flow dynamics in supraventricular tachycardia, frequent heartbeat and premature beat and especially premature ventricular contractions), atrial fibrillation, primary cardiomyopathy and valvular heart disease; 6) uncontrolled or mismanaged blood pressure and blood glucose; 7) cardiovascular disease that had been treated with acupuncture within recent three months; or 8) undergoing other clinical trials.

### Recruitment procedures

The following scheme will be used to recruit participants with angina pectoris. Patients visiting the outpatient clinics and inpatient department in every clinical center will be informed by the cardiologist of the present trial. Research assistants will continue the enrollment process by screening eligible participants according to the inclusion/exclusion criteria. Potential patients in local communities and out-hospital clinics will be recruited by advertisements in the post, leaflets, TV broadcast and newspapers. Participants will be included only if they meet the inclusion criteria and provide written informed consent indicating that they agree to every procedure of the trial and join in the trial willingly.

#### Interventions

In order to ensure the safety of participants, fulfill ethic necessities and improve the prognosis of patients with angina pectoris, we adhered to the European and Chinese Guidelines for the management of patients with chronic stable angina recommendation [37,38]. All participants in the four groups will receive same basic treatment. In addition, according to the guidelines and clinical conditions in China, we shall prescribe antianginal drugs for patients suffering acute angina attack.

The initial acupuncture treatment scheme originates from the clinical practice of TCM. The final scheme was discussed and revised according to advice of clinical acupuncture experts who were consulted in China. Participants in the acupuncture groups will receive 12 sessions of acupuncture treatment over 4 weeks. In each session, participants in all groups except for WL will receive acupuncture treatment bilaterally three times per week and each session will last for 30 min. Each group shares the same basic treatment including health education and basic drug therapy. The whole study period is 20 weeks including a 4-week baseline period, a 4-week treatment period and a 12-week follow-up. We required each participant to record the details of each angina attack and remission in angina diaries. The angina diaries should be kept from baseline to 12 weeks after randomization. All outcomes will be assessed at baseline period and in the 4th, 8th, 12th and 16th weeks after randomization, according to the diaries and related checks.

### Basic treatment

Basic treatment includes health education and primary drugs. We recommend lifestyle modification including increasing exercise, limiting alcohol consumption, weight loss, quitting smoking, *etcetera* for all patients in health education. Basic medication includes aspirin (100 mg once a day); metoprolol (25 mg twice a day); ramipril (5 mg once a day); and atorvastatin (20 mg once every night) [37,38]. Basic treatment lasted from baseline to the completion of the follow-up period.

### Antianginal therapy

Antianginal therapy includes nitroglycerin, nifedipine tablets and suxiao jiuxin wan [39]. In emergency cases of angina attack, participants will be instructed to administer one kind of medicine according to previous treatment history and personal contraindication. Basically, for all patients, we recommend nitroglycerin. Regardless of the type of medicine, participants are required to carefully record the details of medicine, including name, administration time and dosage. Researchers will provide these three drugs to standardize the basic treatment for free: Nitroglycerin (Beijing Yimin Pharmaceutical Co., Ltd., Beijing, China) with State Food and Drugs Administration (SFDA) (China) registration number (H11021022), sublingual dose of 0.5 mg (one tablet); Nifedipine Tablets (CSPC Pharmaceutical Group Limited, Shijiazhuang, China, SFDA: H13021315), oral dose of 10 mg (one tablet); Suxiao Jiuxin pills (SX) (Zhongxin Pharma Tianjin No. 6 Traditional Chinese Medicine Factory, Tianjin, China, SFDA: Z12020025), sublingual dose of 5 to 10 pills. Other antianginal drugs would be considered to violate the study protocol, for which the patient would be eliminated.

There will be one treatment group receiving acupuncture stimulation at acupoints on the disease-affected meridian (DAM), and three different control groups undergoing acupuncture stimulation at acupoints on a non-affected meridian (NAM), non-acupoints (NA) and no intervention, respectively, in addition to routine care. The location and needling methods for acupoints and non-acupoints are demonstrated in Table 2. The name/code and location of the acupoints are consistent with the WHO standards [40].

**Table 2:** Details of each group

**Group**	**Interventions**	**Acupoints**	**Manipulation**
Acupoint on disease- affected meridian	Acupuncture and basic treatment	Neiguan (PC6)	Both points are punctured bilaterally and perpendicularly 2 to 4 cm
		Tongli (HT5)	
Acupoint on non-affected meridian	Acupuncture and basic treatment	Taiyuan (LU9)	Both points are punctured bilaterally and perpendicularly 2 to 4 cm.
		Kongzui (LU6)	
		1) On the front arm of deltoid muscle and biceps brachi junction.	
Non-acupoint	Acupuncture and basic treatment	2) On the ulnar side of the arm, half way between the epicondylus medialis of the humerus and the ulnar side of the wrist.	Both points are punctured bilaterally and perpendicularly 3 to 5 cm.
Waiting list	Basic treatment		

#### DAM group

Based on TCM theory, angina pectoris commonly affects the heart and pericardium meridian, and acupoints located on these two meridians are essential components of acupoint prescription for heart diseases in acupuncture clinics. Thus, we selected Neiguan (PC6) and Tongli (HT5) as obligatory acupoints. PC6 has profoundly been regarded as the key acupoint for curing heart and chest disease in Chinese medicine. As clinically indicated, PC6 can improve cardiac function, enhance myocardial contractility, increase coronary artery blood flow and myocardial oxygen supply and relieve angina pectoris [16,41,42]. HT5 functions to calm the spirit and regulate heart rhythm. The combination of HT5 and PC6 are frequently used to treat angina pectoris and arrhythmia clinically [15].

#### NAM group

In this group, we selected Taiyuan (LU9) and Kongzui (LU6), both of which belong to Lung Meridian of Hand Taiyin. This meridian and its related acupoints in clinical acupuncture practice are not the preferred choice for treating angina pectoris [43]. Nevertheless, they are located inside the forearm, which makes it a suitable control because acupoints chosen in the DAM group are located in the same body area.

#### NA group

We will provide non-acupoint acupuncture treatment for patients in the NA group, in which pre-validated sham acupoints [44] and real insertion of acupuncture needles at bilateral non-acupoints will be administrated, but without achieving a ‘deqi’ sensation.

#### WL group

Participants in this group will receive no acupuncture-related intervention while the trial is in process, but will receive a free 12-session acupuncture treatment over 4 weeks after the completion of the study.

The doctors performing all treatment procedures have at least 5-year experience of acupuncture treatment and a TCM license. All acupoints will be punctured with disposable stainless steel needles (0.25 mm × 40 mm; 0.25 mm × 25 mm; Suzhou Huatuo Medical Appliance Co., Ltd., Suzhou City, China). The needles will be manipulated in a lifting and thrusting technique combined with twirling and rotating manner until the patient feels numbness or other acupuncture sensation (known as ‘deqi’). Then, an auxiliary needle (0.18 mm × 13 mm) will be inserted to 2 mm away from the acupuncture needle to a depth of 2 mm. No manipulation will be delivered to the auxiliary needle. Acupuncture needles and auxiliary needles will be separately connected to an electrode-powered by HANS-200A stimulator (Nanjing Jisheng Medical Technology Company, Nanjing city, China), to induce stimulation to further activate acupoint for 30 min with 2 Hz, rarefaction wave. The electrical stimulation intensity will be adjusted from 0.1 mA to 2.0 mA to make the patients feel comfortable. After retaining for 30 min, all needles will be withdrawn with clean cotton balls pressed to the skin to prevent bleeding.

### Outcome measurement

The primary outcome in this trial is the change from baseline in frequency of angina attack at 4 months. The primary endpoint will be measured 1 day before the inclusion, and the 1st day of the 4th, 8th, 12th and 16th weeks after inclusion.

The secondary outcomes include the following eight items: 1) pain severity of angina (VAS Score); 2) reduction of the dosage of nitroglycerin or Suxiao Jiuxin wan; 3) improvement of exercise capacity assessed by Six minutes’ walk test; 4) changes of ST-T in dynamic ECG; 5) score of Seattle Angina Questionnaire (SAQ); 6) self-rating anxiety scale (SAS); 7) self-rating depression scale (SDS); and 8) incidence of cardiovascular event during the 4 months (developing into unstable angina, acute myocardial infarction and even death).

The six minutes’ walk test and dynamic ECG will be simultaneously measured 1 day before and in the 4th week after inclusion. Others endpoints are measured 1 day before and in the 4th, 8th, 12th and 16th weeks after inclusion.

#### Practitioners Training and quality control

All researchers involved in this trial, all clinical physicians who enroll participants and all assessors who collect data must attend training classes to make sure all practices at each hospital are generalized and standardized. There are both theoretical and practical courses in the training classes. All practitioners finishing the training courses will be accredited a trial certificate by passing the examination.

In order to guarantee the quality of trial process, quality monitoring will be carried out by the CIMS every 3 months, and specially trained physicians will inspect and document the details of the entire trial process.

### Safety and adverse event

For the sake of patient safety, prevention measures and emergency medical plans will include well-equipped treatment rooms, emergency department, cardiovascular specialist and first-line clinical physicians. All adverse events (AEs) associated with acupuncture would be recorded during the treatment and the follow-ups; these AEs include bleeding, hematoma, fainting, serious pain, local infection, *etcetera*. On the other hand, AEs commonly associated with the anti-anginal drugs (basic therapy) will be documented as well, (for example, headaches, dizziness, nausea, flushing, abdominal pain, *etcetera*) [45]. Serious adverse events (SAE) are defined as death or life-threatening events, which may require inpatient hospitalization, cause prolongation of existing hospitalization, or even result in persistent or significant disability/incapacity and need intervention to prevent permanent impairment or damage. If participants suffer any AE/SAE, all details will be documented and reported. Furthermore, SAE will be reported to the principal investigator and the ethics committee immediately so that they can make a decision on whether the patient should withdraw from the trial.

### Sample size calculation and statistical analysis

The sample size calculation was based on a previously study by Richter A. *et al*. [8]. According to which, the clinical effect difference value of the two groups was 4.5. In this study, we incorporated the early clinical pretest, the difference of clinical effect for frequency of weekly angina attacks (DAM and NA) was estimated to be 4.2. Standard deviation for each of the four groups was 8.5 times (α = 0.05; 1-β = 0.90). According to the estimation with NQuery Advisor (Version 4.0, Statistical Solutions Ltd, Ireland), in the bilateral testing, 352 cases are required in this study with 88 cases for each group.

Considering a 15% dropout, therefore, 404 participants in sum should be included in this trial with 101 for each group.

Statistical analysis will be performed by a statistician blinded to the whole trial process using SAS (Version 9.1, SAS Institute Inc, USA) statistical Software packages in the CIMS. For the evaluation of curative effect in this trial, the per-protocol set (PPS) was used. The full analysis set (FAS) was determined according to intention-to-treat population (ITT) and all the patients who were randomized and received at least one treatment session were included in the analysis set. The per-protocol (PPS) population was defined as the patients who completed the study and did not have major protocol violations. Demographic data and other basic indicators were analyzed to measure the balance of the four groups in the baseline. The main indicators and global indicators were analyzed with FAS and PPS. We described the results with the mean, standard deviation, median, P25, P75, maximum and minimum values of the differences between the treatment period and the baseline period. So that a multicenter clinical trial can be adopted in the study, the central effect on the efficacy indicators shall be considered in the analysis. During the comparison of the various groups, other factors that might affect the efficacy will be considered as co-variants for covariance analysis or logistic regression analysis. Missing data will be replaced according to the principle of multiple imputation.

## Discussion

This study potentially may confirm that acupuncture is effective for CSAP in addition to routine care. Our study may further confirm meridian-based acupoint specificity. In the present study, we establish one treatment group and three control groups in this randomized controlled trial. Over the past 20 years, strategies for clinical acupuncture controls fall under three categories: 1) sham acupuncture, in which the skin is punctured with real acupuncture needles either fully at non-acupoint locations or shallowly at acupoint locations; 2) placebo acupuncture, which utilizes non-penetrating acupuncture devices; and 3) wait list and ‘treatment as usual’ [46]. In this study we have adopted both sham acupuncture and wait list controls. Our aim for establishing those two groups is to differentiate and isolate the placebo effect and patient expectancy [47,48] in particular, to further validate the pure effect of acupuncture.

Moreover, the reason we chose acupoints on the Lung meridian in the NAM group as a control to the treatment group is that 1) previous studies have paid close attention to comparisons of the power of real-acupoint with sham-acupoint, however no clinical trials in CSAP have focused on the comparison between acupoints on different meridians; 2) Lung meridian acupoints selected in the NAM group of this trial are located at the upper anterior extremity the superficial acupoint shares the same anatomical tissue as the brachial plexus nerve acupoints of the DAM group. It is hypothesized that by this manner, the effect of NAM could control that of DAM; 3) acupoints on the lung meridian, according to traditional acupuncture theory, are not closely related and are not frequently prescribed for heart diseases, which made it a sub-optimal choice for CSAP. Thus, selecting the acupoints on heart meridian which is believed to be most closely related and indispensable to heart disorders [43], compared with the Lung meridian, is extremely meaningful and helpful in optimizing acupuncture prescription for CSAP.

In conclusion, this trial will provide new and relatively high quality evidence in acupuncture treatment for CSAP. Moreover, this trial may further validate the meridian-based characteristics of acupoint specificity by comparing the strength of acupoints on disease-the affected meridian versus that of a non-affected meridian, to further inspire optimization of acupuncture therapy for CSAP.

## Trial status

The trial is currently recruiting patients.

## Abbreviations

DAM: disease-affected meridian; AE: adverse events; NAM: non-affected meridian; CSAP: chronic stable angina pectoris; FAS: full analysis set; ITT: intention-to-treat population; NA: non-acupoint; PPS: per-protocol set; RCT: randomized controlled trial; SAE: serious adverse events; SAQ: Seattle Angina Questionnaire; SAS: self-rating anxiety scale; SDS: self-rating depression scale; SMS: short message service; TCM: traditional Chinese medicine; WL: wait-listed.

## Competing interests

The authors declare that they have no competing interests.

## Authors’ contributions

DHL and MXY contributed equally to this article. DHL, MXY, LZ, HZ, YL, JLL, JYL, JL and FRL participated in the conception and design of the trial, planning the analysis of the data and drafting the manuscript. XRC, RHW, JC, JS participated in data collection and in charge of recruitment and treatment of patients in each center. All the authors read, discussed, revised and approved the final manuscript.
